# The Role of Balanced Training and Testing Data Sets for Binary Classifiers in Bioinformatics

**DOI:** 10.1371/journal.pone.0067863

**Published:** 2013-07-09

**Authors:** Qiong Wei, Roland L. Dunbrack

**Affiliations:** Institute for Cancer Research, Fox Chase Cancer Center, Philadelphia, Pennsylvania, United States of America; Miami University, United States of America

## Abstract

Training and testing of conventional machine learning models on binary classification problems depend on the proportions of the two outcomes in the relevant data sets. This may be especially important in practical terms when real-world applications of the classifier are either highly imbalanced or occur in unknown proportions. Intuitively, it may seem sensible to train machine learning models on data similar to the target data in terms of proportions of the two binary outcomes. However, we show that this is not the case using the example of prediction of deleterious and neutral phenotypes of human missense mutations in human genome data, for which the proportion of the binary outcome is unknown. Our results indicate that using balanced training data (50% neutral and 50% deleterious) results in the highest balanced accuracy (the average of True Positive Rate and True Negative Rate), Matthews correlation coefficient, and area under ROC curves, no matter what the proportions of the two phenotypes are in the testing data. Besides balancing the data by undersampling the majority class, other techniques in machine learning include oversampling the minority class, interpolating minority-class data points and various penalties for misclassifying the minority class. However, these techniques are not commonly used in either the missense phenotype prediction problem or in the prediction of disordered residues in proteins, where the imbalance problem is substantial. The appropriate approach depends on the amount of available data and the specific problem at hand.

## Introduction

In several areas of bioinformatics, binary classifiers are common tools that have been developed for applications in the biological community. Based on input or calculated feature data, the classifiers predict the probability of a positive (or negative) outcome with probability *P*(+) = 1–*P*(–). Examples of this kind of classifier in bioinformatics include the prediction of the phenotypes of missense mutations in the human genome [1]–[8], the prediction of disordered residues in proteins [9]–[17], and the presence/absence of beta turn, regular secondary structures, and transmembrane helices in proteins [18]–[21].

While studying the nature of sequence and structure features for predicting the phenotypes of missense mutations [22]–[25], we were confronted by the fact that we do not necessarily know the rate of actual deleterious phenotypes in human genome sequence data. Recently, very large amounts of such data have become available, especially from cancer genome projects comparing tumor and non-tumor samples [26]. This led us to question the nature of our training and testing data sets, and how the proportions of positive and negative data points would affect our results. If we trained a classifier with balanced data sets (50% deleterious, 50% neutral), but ultimately genomic data have much lower rates of deleterious mutations would we overpredict deleterious phenotypes? Or should we try to create training data that resembles the potential application data? Should we choose neutral data that closely resembles potential input, for example human missense mutations in SwissVar, or should we use more distinct, for example data from close orthologues of human sequences in other organisms, in particular primates?

Traditional learning methods are designed primarily for balanced data sets. The most commonly used classification algorithms such as Support Vector Machines (SVM), neural networks and decision trees aim to optimize their objective functions that usually lead to the maximum overall accuracy – the ratio of the number of true predictions out of all predictions made. When these methods are trained on very imbalanced data sets, they often tend to produce majority classifiers – over-predicting the presence of the majority class. For a majority positive training data set, these methods will have a high true positive rate (TPR) but a low true negative rate (TNR). Many studies have shown that for several base classifiers, a balanced data set provides improved overall classification performance compared to an imbalanced data set [27]–[29].

There are several methods in machine learning for dealing with imbalanced data sets such as random undersampling and oversampling [29], [30], informed undersampling [31], generating synthetic (interpolated) data [32], [33], sampling with data cleaning techniques [34], cluster-based sampling [35] and cost-sensitive learning in which there is an additional cost to misclassifying a minority class member compared to a majority class member [36], [37]. Provost has given a general overview of machine learning from imbalanced data sets [38], and He and Garcia [39] show the major opportunities, challenges and potential important research directions for learning from imbalanced data.

Despite the significant literature in machine learning from imbalanced data sets, this issue is infrequently discussed in the bioinformatics literature. In the missense mutation prediction field, training and testing data are frequently not balanced and the methods developed in machine learning for dealing with imbalanced data are not utilized. Table 1 shows the number of mutations and the percentage of deleterious mutations in training data set and testing data set for 11 publicly available servers for missense phenotype prediction [1]–[3], [6], [7], [40]–[42]. Most of them were trained on imbalanced data sets, especially, nsSNPAnalyzer [3], PMut [2], [43], [44], SeqProfCod [41], [45] and MuStab [46]. With a few exceptions, the balanced or imbalanced nature of the training and testing set in phenotype prediction was not discussed in the relevant publications. In one exception, Dobson et al. [47] determined that measures of prediction performance are greatly affected by the level of imbalance in the training data set. They found that the use of balanced training data sets increases the phenotype prediction accuracy compared to imbalanced data sets as measured by the Matthews Correlation Coefficient (MCC). The developers of the web servers SNAP [5], [6] and MuD [7] also employed balanced training data sets, citing the work of Dobson et al. [47].

**Table 1:** The #mutations and percentage of deleterious mutations for published methods.

Program	Training data	#mutations	%D	Testing data	#mutations	%D	ACC	BACC
SNAP	PMD/EC dataset^a^	80817	51	CrossValidation	80817	51	80	80
SeqProfCod	SP-Dec05^b^	8987	69	SP-Dec06^c^	2008	40	73	69
SNPs3D-profile	HGMD disease and inter-orthologresidue difference	21246	45	CrossValidation	21246	45	86	85
SNPs3D-stability	HGMD disease and inter-orthologresidue difference with structureinformation	6077	62	CrossValidation	6077	62	78	80
PMut	SWP-Lac^d^	11588	81	CrossValidation	11588	81	87	92
	SWP-Evol^e^	20706	45	CrossValidation	20706	45	84	81
	PDBst^f^	2207	60	CrossValidation	2207	60	87	86
PHD-SNP	HumVar	21185	61	CrossValidation	21185	61	74	73
	HumVarProf	8718	61	NewHumVar	935	16	74	74
				OutPhD-SNP08^g^	34314	50	76	76
nsSNPAnalyzer	SwissVar database> = 10 homologous sequence	4013	87	SwissVar database<10 homologous sequence	205	85	75	73
SeqSubPred	The mutations from Swiss-Protdatabase (released version 57.2)	49532	41	SP-Dec06	2008	40	80	79
MuD	Bromberg and Rost data set withstructure information^h^	12133	51	LacI	4041	44	81	80
				HIV-1 protease	336	67	69	70
				T4 Lysozyme	2015	32	47	67
MuStab	Data set from PhD-SNP	1480	31	CrossValidation	1480	31	85	81
PolyPhen2	HumDiv^i^	9476	33	CrossValidation	9476	33	84	86
	HumVar^j^	21978	59	CrossValidation	21978	59	76	77

a 39887 disease mutations from PMD database, 13990 neutral mutations from PMD and 26840 neutral mutations from residues that differed in pairwise alignments of enzymes with experimentally annotated similarity in function and the same EC numbers.

b Derived from the Swiss-Prot release 48 (Dec 2005).

c Includes only mutations from protein sequence deposited in Swiss-Prot from January to November 2006 (release 51).

d The neutral mutations are extracted from LacI.

e The neutral mutations are extracted from the evolutionary model.

f Structure-based case.

g Available at http://gpcr2.biocomp.unibo.it/~emidio/PhD-SNP/OutPhD-SNP08.txt.

h The data set of SNAP.

i 3155 damaging alleles annotated in the Uniprot database as causing human Mendelian diseases and affecting protein stability or function, 6321 differences between human proteins and their closely related mammalian homologs, assumed to be nondamaging.

j 13032 human disease-causing mutations from UniProt and 8946 human nonsynonymous single-nucleotide polymorphisms without annotated involvement in disease.

The sources of deleterious and neutral mutation data are also of some concern. These are also listed in Table 1 for several available programs. The largest publicly available data set of disease-associated (or deleterious) mutations is the SwissVar database [48]. Data in SwissVar are derived from annotations in the UniprotKB database [49]. Care et al. assessed the effect of choosing different sources for neutral data sets [50], including SwissVar human polymorphisms for which phenotypes are unknown, sequence differences between human and mammalian orthologues, and the neutral variants in the Lac repressor [51] and lysozyme data sets [52]. They argue that the SwissVar human polymorphism data set is closer to what one would expect from random mutations under no selection pressure, and therefore represent the best “neutral” data set. They show convincingly that the possible accuracy one may achieve depends on the choice of neutral data set.

In this paper, we investigate two methodological aspects of the binary classification problem. First, we consider the general problem of what effect the proportion of positive and negative cases in the training and testing sets has on the performance as assessed by some commonly used metrics. The basic question is how to achieve the best results, especially in the case where the proportion in future applications of the classifier is unknown. We show that the best results are obtained when training on balanced data sets, regardless of the rate of proportions of positives and negatives in the testing set. This is true as long as the method of assessment on the testing set appropriately accounts for any imbalance in the testing set. Our results indicate that “balanced accuracy” (the mean of TPR and TNR) is quite flat with respect to testing proportions, but is quite sensitive to balance in the training set, reaching a maximum for balanced training sets. The Matthews’ correlation coefficient is sensitive to the proportions in both the testing set and the training set, while the area under the ROC curve is not very sensitive to the testing set proportions and also not to the training set proportions when the minority class is at least 30% of the training data. Thus, while the testing measures depend to greater or lesser extents on the balance of the training and/or testing sets, they all achieve the best results on the combined use of balanced training sets and balanced testing sets.

Second, for the specific case of missense mutations, we show data that mutations derived from human/non-human-primate sequence comparisons may provide a better data set compared to the human polymorphism data. This is precisely because the primate sequence differences with human proteins are more consistent with what we would expect on biophysical grounds than the human variants. The latter are of unknown phenotype and may be the result of recent mutations in the human genome, some of which may be at least mildly to moderately deleterious.

## Methods

### Data Sets

To compile a human mutation data set, we downloaded data on mutations from the SwissVar database (release 57.8 of 22–Sep-2009) [48]. After removing unclassified variants, variants in very long proteins to reduce computation time (sequences of more than 2000 amino acids), redundant variants, and variants that are not accessible by single-site nucleotide substitutions (just 150 mutation types are accessible by single-site nucleotide change), we compiled separate human disease mutation as the deleterious mutations and human polymorphism as the neutral mutations, these two data sets labeled *HumanDisease* and *HumanPoly* respectively.

Non-human primate sequences were obtained from UniprotKB [49]. We used PSI-BLAST [53], [54] to identify likely primate orthologues of human proteins in the SwissVar data sets using a sequence identity cutoff of 90% between the human and primate sequences. More than 75% of the human-primate pairs we identified in this procedure have sequence identity greater than 95%, and are very probably orthologues. Mutations without insertions or deletions within 10 amino acids on either side of the mutation of amino acid differences in the PSI-BLAST alignments were compiled into a data set of human/primate sequence differences, *PrimateMut*. Only those single-site nucleotide substitutions were included in *PrimateMut*, although we did not directly check DNA sequences to see if this is how the sequence changes occurred. Finally, where possible, we mapped the human mutation sites in the *HumanDisease*, *HumanPoly*, and *PrimateMut* data sets to known structures of human proteins in the PDB using SIFTS [55], which provides Uniprot sequence identifiers and sequence positions for residues in the PDB. This mapping produced three data sets, *HumanDiseaseStr*, *HumanPolyStr*, and *PrimateMutStr.*

To produce an independent test set, we compared the SwissVar release 2012_03 of March 21, 2012 with that of release 57.8 of Sep. 22, 2009 used in the previous calculations. We selected the human-disease mutations and human polymorphisms contained in the new release and searched all human proteins in Uniprot/SwissProt against primate sequences to get additional primate polymorphisms, and then compared these human disease mutations and primate polymorphisms with our training data set to get those human disease mutations and primate polymotphisms not contained in the training data set as our independent testing data set. The resulting independent testing data set contains 2316 primate polymorphisms, 1407 human polymorphisms and 1405 human disease mutations.

The data sets are available in Data S1.

### Calculation of Sequence and Structure Features

We used PSI-BLAST [53], [54] to search human and primate protein sequences against the database UniRef90 [49] for two rounds with an E-value cutoff of 10 to calculate the PSSM score for the mutations. From the position-specific scoring matrices (PSSMs) output by PSI-BLAST, we obtained the dPSSM score which is the difference between the PSSM score of the wildtype residues and the PSSM scores of the mutant residues.

To calculate a conservation score, we parsed the PSI-BLAST output to select homologues with sequence identity greater than 20% for each human and primate protein. We used BLASTCLUST to cluster the homologues of each query using a threshold of 35%, so that the sequences in each cluster were all homologous to each other wither a sequence identity ≥35%. A multiple sequence alignment of the sequences in the cluster containing the query was created with the program Muscle [56], [57]. Finally, the multiple sequence alignment was input to the program AL2CO [58] to calculate the conservation score for human and primate proteins.

For each human mutation position, we determined if the amino acid was present in the coordinates of the associated structures (according to SIFTS). Similarly, for each primate mutation, we determined whether the amino acid of the human query homologue was present in the PDB structures. For each protein in our human and primate data sets whose (human) structure was available in the PDB according to SIFTS, we obtained the symmetry operators for creating the biological assemblies from the PISA website and applied these symmetry operators to create coordinates for their predicted biological assemblies. We used the program Naccess [59] to calculate surface area for each wildtype position in the biological assemblies as well as in the monomer chains containing the mutation site (i.e., from coordinate files containing only a single protein with no biological assembly partners or ligands). For the human mutation position, if the amino acid can be presented in the coordinates of more than one associated structures, we calculated the surface area for those associated structures and get the minimal surface area as the surface area of that human mutation.

### Contingency Tables for Mutations

We compared the different data sets using a *G*-test, for which the commonly used Chi-squared test [60] is only an approximation (both developed by Pearson in 1900 [61]; Chi-squared was developed by Pearson because logarithms were time-consuming to calculate),
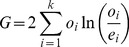
(1)where *o_i_* is the observed number of category *i* and *e_i_* is the expected number of category *i*, *k* is the total number of categories. *G* is sometimes called *G* ^2^ by mistaken analogy to 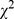.

Assuming *N_i_* denotes the number of mutations in data set 1 and *N*_2_ denotes the number of mutations in data set 2 and for each type of mutation, *i*, *o*_1_(*i*) is the observed number of mutation *i* in data set 1 and *o*_2_(*i*) is the observed number of mutation *i* in data set 2, then the total frequency of mutation *i* across both data sets is 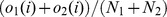. We calculate the expected number of mutations of type *i* in data set 1 and 2:
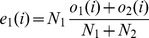
(2)

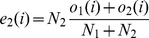
(3)


So *G* for those two data sets is:
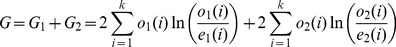
(4)


Because the two sets of data are independent and being compared to their average, there are 2*k*-1 degrees of freedom (299 for 150 mutations accessible by single-nucleotide mutations).

### Accuracy Measures

We focus on the question of which measure is appropriate to evaluate the performance of SVM models depending on whether the training or testing sets are imbalanced. We define several of these measures as follows. The true positive rate (TPR) measures the proportion of actual positives which are correctly identified. The true negative rate (TNR) measures the proportion of actual negatives which are correctly identified. Positive predictive value is defined as the proportion of the true positive against all the positive results (both true positives and false positives) and the overall accuracy is the proportion of true results (both true positives and true negatives) in the population. These measures are defined as:
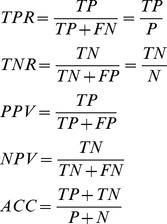
(5)where *P* is the number of positive examples and *N* is the number of negative examples in the testing data set, *TP* is the number of true positives, *TN* is the number of true negatives, *FP* is the number of false positives and *FN* is the number of false negatives.

When the testing data are highly imbalanced, it is easy to achieve high accuracy (ACC) simply by predicting every testing data point as the majority class. To evaluate the performance of an SVM model on imbalanced testing sets, we use three measures: Balanced Accuracy (BACC) [62], which avoids inflated performance estimates on imbalanced data sets, the Matthews Correlation Coefficient (MCC) [63] which is generally regarded as a balanced measure, and the area under Receiver Operating Characteristic (ROC) curves (AUC) [64]. The balanced accuracy and Matthews Correlation Coefficient are defined as:
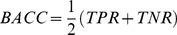
(6)

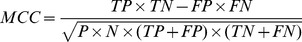
(7)


The ROC curve is a plot of the true positive rate versus the false positive rate for a given predictor. A random predictor would give a value of 0.5 for the area under the ROC curve, and a perfect predictor would give 1.0. The area measures discrimination, that is, the ability of the prediction score to correctly sort positive and negative cases.

## Results

### The Selection of Neutral Data Sets

From SwissVar, we obtained a set of human missense mutations associated with disease and a set of polymorphisms of unknown phenotype, often presumed to be neutral. From the same set of proteins in SwissVar, we identified single-site mutations between human proteins and orthologous primate sequences with PSI-BLAST (see Methods). Table 2 gives the number of proteins and mutations in each of six data sets: *HumanPoly, HumanDisease, PrimateMut* and those subsets observable in experimental three-dimensional structures of the human proteins, *HumanPolyStr, HumanDiseaseStr,* and *PrimateMutStr*.

**Table 2:** The number of proteins, mutations and self G-square for each data set.

Data set*	#Proteins	Num	G_1_	G_2_	G
*HumanPoly*	10619	29467	80.3	82.7	163.0
*HumanDisease*	2446	19056	64.6	63.5	128.2
*PrimateMut*	3153	22790	84.3	84.4	168.6
*HumanPolyStr*	1302	3325	77.0	87.4	164.3
*HumanDiseaseStr*	562	6938	69.7	79.8	149.4
*PrimateMutStr*	719	3575	78.9	85.6	164.5

* Data sets are available in Supplemental Material.

We decided first to evaluate whether *HumanPoly* or *PrimateMut* would make a better set of neutral mutations for predicting the phenotype of human missense mutations. We were especially concerned that the phenotypes of the *HumanPoly* mutations are unknown. We use the value of *G*, for which 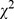 is only an approximation [60], to compare the distribution of those single-nucleotide mutations in the different data sets. *G* compares a set of observed counts with a set of expected counts over discrete categories, such as the possible single-site mutations. To compare two different data sets, we calculated the expected counts for each data set using frequencies from the combined data sets and then calculated *G = G*_1_+*G*_2_ (*G*_1_ for data set 1 and *G*_2_ for data set 2).

To see how *G* behaves, we calculated *G* for each of the six data sets by randomly splitting each into two subsets and then calculating the observed numbers, expected numbers and *G* for 150 mutation types (those accessible by single-nucleotide mutations) using Equations 2, 3 and 4. Table 2 shows *G* for the six data sets. The *P*-values for these values of *G*, calculated from 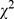 tables with 299 degrees of freedom, are all equal to 1.0, demonstrating that the half subsets are quite similar to each other as expected.

By contrast, the values of *G* when comparing two different data sets exhibit much larger values. Table 3 shows *G* for various pairs of data sets. According to the *G* values in Table 3, the large data sets *HumanPoly* and *PrimateMut* are the most similar, while *HumanDisease* is quite different from either. However, *HumanPoly* is closer to *HumanDisease* than *PrimateMut*, which brings up the question of which is the better neutral data set. The values of *G* for the subsets with structure follow a similar pattern (Table 3). P-values for the values of *G* in Table 3 are all less than 0.001.

**Table 3:** The G values for different datasets against each other.

Data set 1	Data set 2	*N*_1_	*N*_2_	*G*_1_	*G*_2_	*G*
*HumanPoly*	*HumanDisease*	29467	19056	3023.1	1976.8	4999.9
*HumanPoly*	*PrimateMut*	29467	22790	1013.7	1461.5	2475.2
*HumanDisease*	*PrimateMut*	19056	22790	5198.9	4376.4	9575.3
*HumanPolyStr*	*HumanDiseaseStr*	3325	6938	742.8	329.2	1071.9
*HumanPolyStr*	*PrimateMutStr*	3325	3575	288.6	307.0	595.6
*HumanDiseaseStr*	*PrimateMutStr*	6938	3575	807.6	1516.0	2323.6

Care et al. [50] showed that the Swiss-Prot polymorphism data are closer to nucleotide changes in non-coding sequence regions than human/non-human mammal mutations are. However, the non-coding sequences are not under the same selection pressure as coding regions are. While positions with mutations leading to disease are likely to be under strong selective pressure (depending on the nature of the disease), it is still likely that positions of known neutral mutations are under some selection pressure to retain basic biophysical properties of the amino acids at those positions.

**Figure 1:**
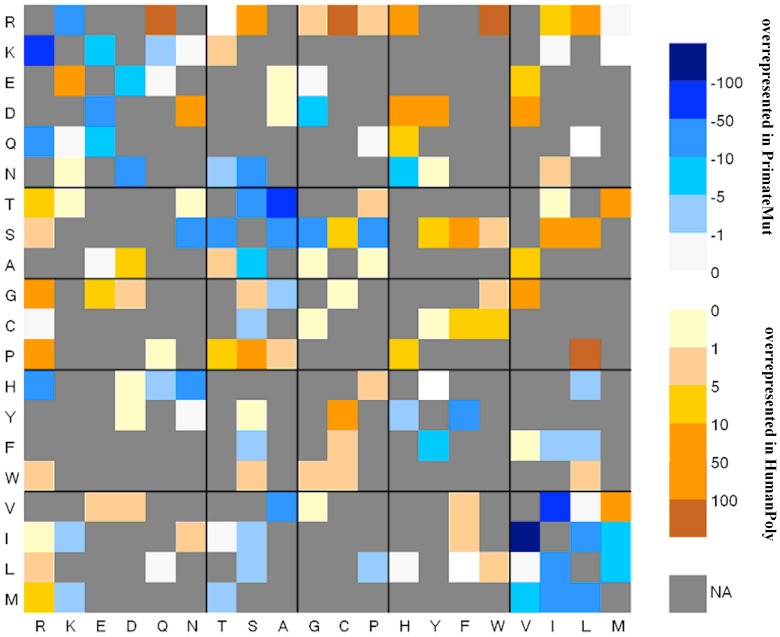
The contributions to G for *HumanPoly* and *PrimateMut*. Only those 150 mutations accessible by single-nucleotide changes are shown in color; others are shown in gray. Wildtype residue types are given along the x-axis and mutant residue types are given along the y-axis. Blue squares indicate substitution types that are overrepresented in *PrimateMut*, while orange squares indicate substitution types that are overrepresented in *HumanPoly*.

To show this, we plotted the contributions to *G* for *HumanPoly* and *PrimateMut* as a heat map in Figure 1. From Equation 4, the contribution for any one mutation is proportional to:
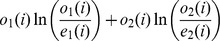



The data set providing overrepresentation of category *i* having a positive value and the data set with an underrepresentation of category *i* having a negative value but with smaller absolute value, so that the sum is always positive. Substitutions with very different frequencies in the two data set contribute much more to *G*. To create a heat map, we plotted the value of:
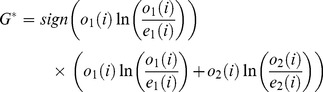
(9)for each mutation type where 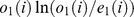 represents the value of mutation *i* in the *HumanPoly* data and 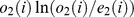 represents the value of mutation *i* in the *PrimateMut* data set. G^*^ is positive (orange colors in Figure 1) when a mutation is overrepresented in the *HumanPoly* data, compared to the *PrimateMut* data. G^*^ is negative (blue colors in Figure 1) when a mutation is overrepresented in the *PrimateMut* data, compared to the *HumanPoly* data.

It is immediately obvious from Figure 1 that mutations we would consider on biophysical grounds to be largely neutral (R→K, F→Y, V→I and vice versa) are overrepresented in the *PrimateMut* data compared to the *HumanPoly* data. Conversely, mutations that on biophysical grounds we would expect to be deleterious (R→W, mutations of C, G, or P to other residue types, large aromatic to charged or polar residues) are overrepresented in the *HumanPoly* data compared to the *PrimateMut* data.

We calculated predicted disorder regions for the proteins in each of the data sets using the programs IUpred [10], Espritz [65], and VSL2 [66]. Residues were predicted to be disordered if two of the three programs predicted disorder. According to predicted disorder regions, we calculated whether the mutation positions in each data set were in regions predicted to be ordered or disordered. In the *HumanPoly* and *PrimateMut* data sets, 31% and 23.6% of the mutations were predicted to be in disordered regions respectively, while in the *HumanDisease* set only 14.3% of the mutations were in predicted disordered regions. Thus, the differences between *HumanPoly* and *PrimateMut* are not due to differences in one important factor that may lead to additional mutability of amino acids, in that disordered regions are more highly divergent in sequence than folded protein domains. This result does explain why the proportion of residues in *HumanDisease* that can be found in known structures (*HumanDiseaseStr*), 36.4%, is so much higher than that for *HumanPoly* and *PrimateMut*, 11.3% and 15.7% respectively.

Further, we checked if the proteins in the different sets had different numbers of homologues in Uniref100, considering that the disease-related proteins may occur in more conserved pathways in a variety of organisms. We calculated the average number of proteins in clusters of sequences related to each protein in the three sets using BLASTCLUST, as described in the Methods. Proteins in each cluster containing a query protein were at least 35% identical to each other and the query. Proteins in the *HumanDisease, HumanPoly,* and *PrimateMut* had 26.4, 25.8, and 28.5 proteins on average respectively (standard deviations of 89.6, 103.2, and 92.0 respectively). Thus the *HumanDisease* proteins are intermediate in nature between the *PrimateMut* and *HumanPoly* proteins in terms of the number of homologues, although the numbers are not substantially different.

It appears then that the *PrimateMut* data show higher selection pressure (due to longer divergence times) for conserving biophysical properties than the *HumanPoly* data. Since polymorphisms among individuals of a species, whether human or primate, are relatively rare, the majority of sequence differences between a single primate’s genome and the reference human genome are likely to be true species differences. Thus, they are likely to be either neutral or specifically selected for in each species. On the other hand, the SwissVar polymorphisms exist specifically because they are variations among individuals of a single species. They are of unknown phenotype, especially if they are not significantly represented in the population. We therefore argue that the *PrimateMut* data are a better representation of neutral mutations than the *HumanPoly* data. In what follows, we use the *PrimateMut* data as the neutral mutation data set, unless otherwise specified.

We calculated two sequence-based and two structure-based features for the mutations in data sets *HumanPolyStr*, *HumanDiseaseStr* and *PrimateMutStr* to compare the prediction of missense phenotypes when the neutral data consists of human polymorphisms or primate sequences. From *HumanDiseaseStr,* we selected a sufficient number of human disease mutations to combine with human polymorphisms (called *Train_HumanPoly*) and primate polymorphisms (called *Train_Primate*) to construct two balanced training data sets. From our independent testing data set (described in the Methods Section), we selected sufficient human disease mutations to combine with human polymorphisms (called *Test_HumanPoly*) and primate polymorphisms (called *test_primate*) to create two balanced independent testing data sets. Table 4 shows the results of SVM model trained by training data sets *Train_humanPloy* and *Train_Primate*, and tested by independent testing data sets *Test_HumanPoly* and *Test_Primate*.

**Table 4:** Performance of the models trained by human polymorphism and primate polymorphism.

Training data	Testing data	TPR	TNR	PPV	NPV	BACC	MCC	AUC
*Train_Primate*	CrossValidation	**84.0**	**78.2**	**79.4**	**83.0**	**81.1**	**0.623**	**0.88**
	*Test_Primate*	82.5	81.7	81.9	82.4	82.1	0.642	0.89
	*Test_HumanPoly*	82.5	67.3	71.6	79.4	74.9	0.504	0.82
*Train_HumanPoly*	CrossValidation	**80.9**	**64.1**	**69.3**	**77.1**	**72.5**	**0.457**	**0.79**
	*Test_Primate*	78.1	82.1	81.4	79.0	80.1	0.603	0.88
	*Test_HumanPoly*	78.1	**70.6**	72.7	76.3	**74.4**	0.489	0.82

The results in Table 4 show that the primate polymorphisms achieve higher cross-validation accuracy than the human polymorphisms on all measures. This confirms that the primate polymorphisms are more distinct in their distribution from the human disease mutations than the human polymorphisms. In particular, the true negative rate for the primate cross-validation results are much higher than for the human polymorphism results. Further, we tested each model (*Train_Primate* and *Train_HumanPoly*) on independent data sets. The two testing data sets, *Test_Primate* and *Test_HumanPoly* contain the same disease mutations but different neutral mutations. The *Train_Primate* model achieves the same TPR for each of the independent testing set at 82.5%, since the disease mutations are the same in each of the testing sets. Similarly, *Train_HumanPoly* achieves the same TPR for each of the testing sets at a lower rate of 78.1% since the human disease mutations are easier to distinguish from the primate mutations than the human polymorphisms. As may be expected, the TNR of *Train_HumanPoly* is better with *Test_HumanPoly* (70.6%) than is *Train_Primate* (67.3%), since the negatives are from similar data sources (human polymorphisms).

It is interesting that regardless of the training data set, the balanced measures of accuracy are relatively similar for a given testing data set. For *Test_Primate*, the BACC is 82.1% and 80.1% for the primate and human training data sets respectively. For *Test_HumanPoly*, the BACC values are 74.9% and 74.4% respectively. The MCC and AUC measures in Table 4 show a similar phenomenon. Thus, the choice of neutral mutations in the testing set has a strong influence on the results, while the choice of the neutral mutations in the training data set less so.

### The Importance of Balanced Training Sets

The more general question we ask is how predictors behave depending on the level of imbalance in either the training set or testing set or both. In the case of missense mutations, we do not a priori know what the deleterious mutation rate may be in human genome data. To examine this, we produced five training data sets (*train_10*, *train_30*, *train_50*, *train_70* and *train_90*) using the same number of training examples, but with a different class distribution ranging from 10% deleterious (*train_10*) to 90% deleterious (*train_90*). We trained SVMs on these data sets using four-features: the difference in PSSM scores between wildtype and mutant residues, a conservation score, and the surface accessibility of residues in biological assemblies and protein monomers.

**Figure 2:**
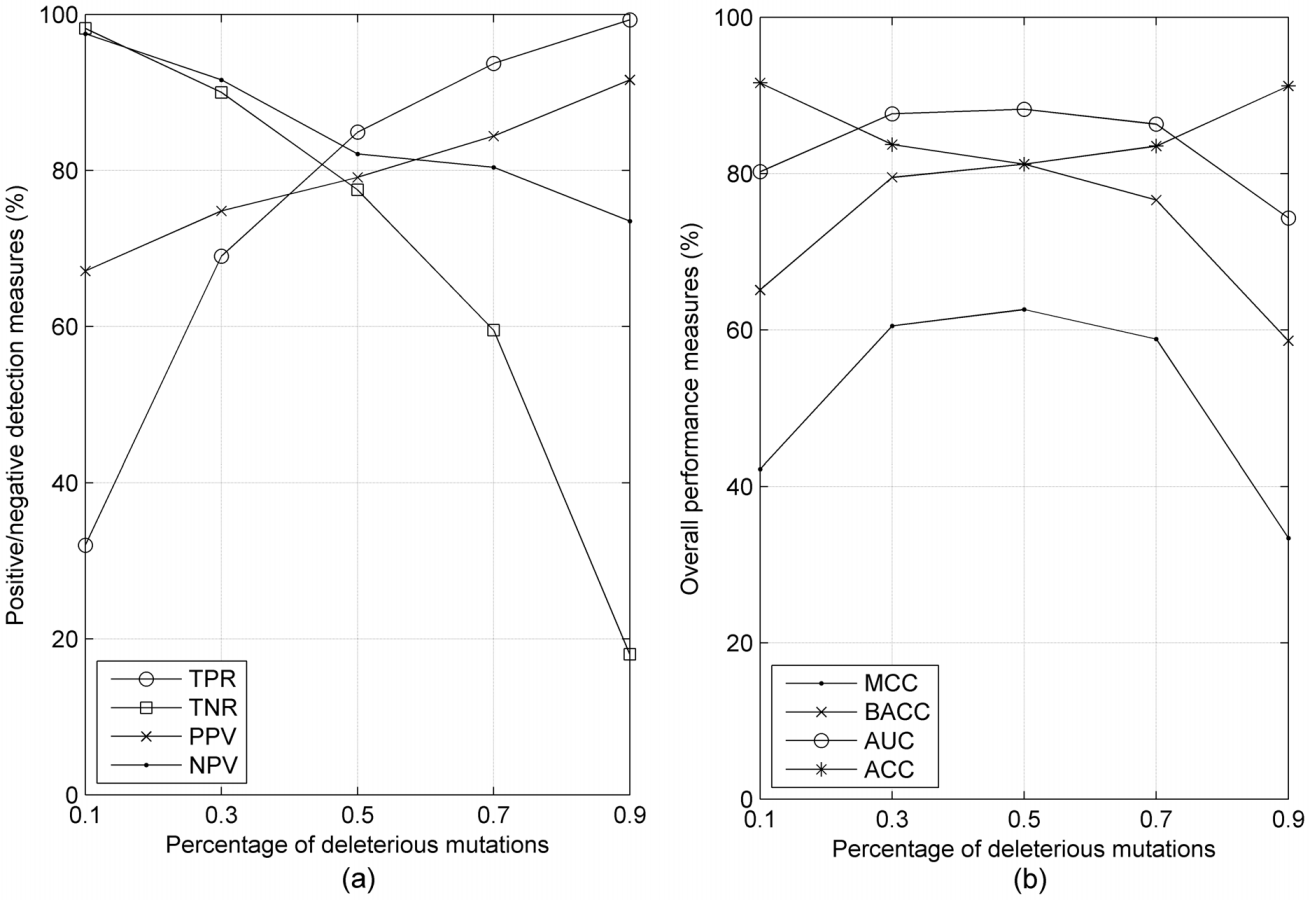
The cross-validation results of five SVM models trained on data sets that are 10%, 30%, 50%, 70% and 90% deleterious mutations (x-axis = 0.1, 0.3, 0.5, 0.7 and 0.9 respectively). (a) Values for TPR, TNR, PPV, and NPV. (b) Values for MCC, BACC, AUC, and ACC.

Figure 2a shows the performance of the five SVM models in 10-fold cross-validation calculations in terms of true positive rate (TPR), true negative rate (TNR), positive predictive value (PPV), and negative predictive value (NPV) as defined in Equation 5. In cross validation, the training and testing sets contain the same frequency of positive and negative data points. Thus on *train_10*, the TPR is very low while the TNR is very high. This is a majority classifier and most predictions are negative. *Train_90* shows a similar pattern but with negatives and positives reversed. The PPV and NPV show a much less drastic variation as a function of the deleterious and neutral content of the data sets. For instance, PPV ranges from about 65% to 90% while TNR ranges from 35% to 100% for the five data sets.

In Figure 2b, we show four measures of accuracy: ACC, BACC, MCC, and AUC. Overall accuracy, ACC, reaches maximum values on the extreme data sets, *train_10* and *train_90.* These data sets have highly divergent values of TPR and TNR as shown in Figure 2a and are essentially majority classifiers. By contrast, the other three measures are designed to account for imbalanced data in the testing data sets. BACC is the mean of TPR and TNR. It achieves the highest result in the balanced data set, *train_50*, and the lowest results for the extreme data sets. The range of BACC is 59% to 81%, which is quite large. Similarly, the MCC and AUC measures also achieve cross-validation maximum values on *train_50* and the lowest values on *train_10* and *train_90*. The balanced accuracy and Matthews Correlation Coefficient are highly correlated, although BACC is a more intuitive measure of accuracy.

**Figure 3:**
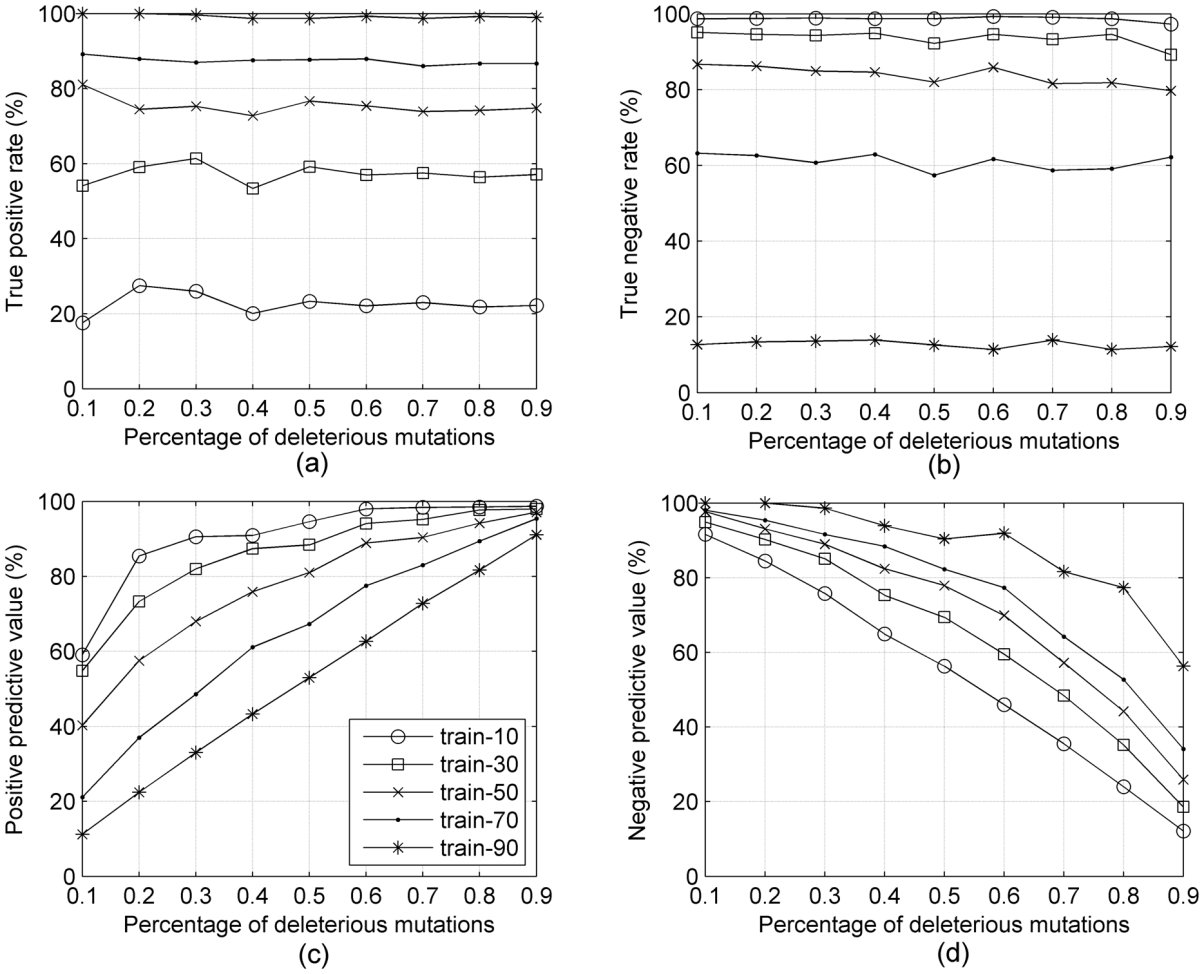
(a) TPR, (b) NPR, (c) PPV, and (d) NPV of five SVM models trained on 5 different data sets (train_10, train_30, train_50, train_70, and train_90) tested by 9 different testing data sets, ranging from 10% deleterious (x-axis = 0.1) to 90% deleterious (x-axis = 0.9).

To explore these results further, we created 9 independent testing data sets using the same number of testing examples, but with different class distribution (the percentage of deleterious mutations from 10%–90%) to test the five SVM models described above (*train_10*, *train_30*, etc.). Figure 3 shows the performance of those five SVM models tested by the 9 different testing data sets.

In Figure 3a and Figure 3b, we show that the true positive and true negative rates are highly dependent on the fraction of positives in the training data set but nearly independent of the fraction of positives in the testing data set. The true positive rate and true negative rate curves of the five SVM models are flat and indicate that the true positive rate and true negative rate are determined by the percentage of the deleterious mutations in the training data – a higher percentage of deleterious mutations in training data leads to a higher true positive rate and a lower true negative rate. Figure 3c shows the positive predictive value which is defined as the proportion of the true positives against all the positive predictions (both true positives and false positives). Figure 3d shows the negative predictive value, which is defined similarly for negative predictions. In both cases, the results are highly correlated with the percentages of positives and negatives in the training data. The curves in Figure 3c show that the positive predictive value of the five SVM models increases with increasing percentage of deleterious (positive) mutations in both the training and testing data sets. The SVM model trained by data set *train_10* achieves the best PPV while Figure 3a shows that this model also has the lowest TPR (less than 30%) for all nine testing data sets, because its number of false positives is very low (it classifies nearly all data points as negative). The NPV results are similar but the order of training sets is reversed and the NPV numbers are positive correlated with the percentage of negative data points in the testing data.

**Figure 4:**
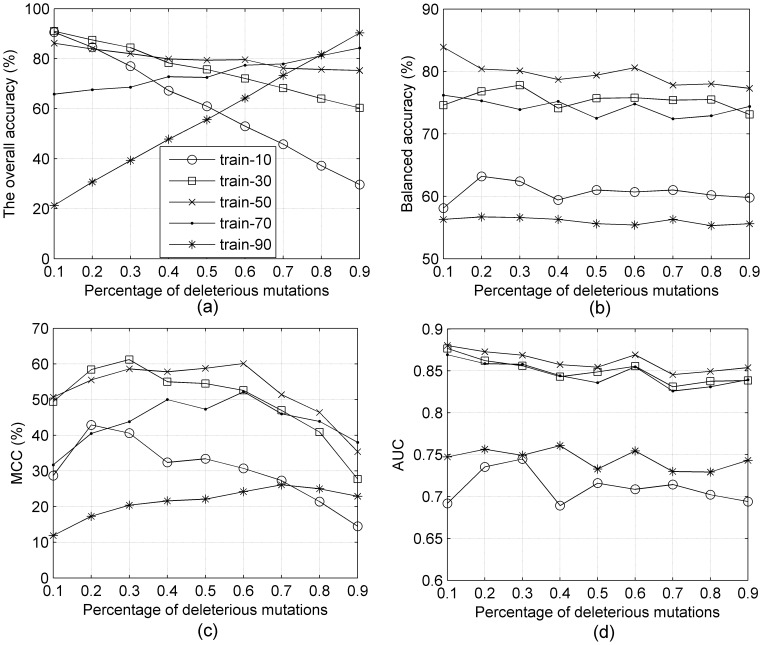
(a) ACC, (b) BACC, (c) MCC, and (d) AUC of five SVM models trained on 5 different data sets (train_10, train_30, train_50, train_70, and train_90) tested by 9 different testing data sets, ranging from 10% deleterious (x-axis = 0.1) to 90% deleterious (x-axis = 0.9).

In Figure 4, we show four measures that assess the overall performance of each training set model on each testing data set – the overall accuracy (ACC) in Figure 4a, the balanced accuracy (BACC) in Figure 4b, the Matthews correlation coefficient (MCC) in Figure 4c, and the area under the ROC curve (AUC) in Figure 4d. The overall shapes of the curves for the different measures are different. The ACC curves, except for *train_50*, are significantly slanted, especially the *train_10* and *train_90* curves. The BACC curves are all quite flat. The MCC curves are all concave down, showing diminished accuracy for imbalanced testing data sets on each end. The AUC curves are basically flat but bumpier than the BACC curves. The figures indicate that the various measures are not equivalent.

The balanced accuracy, BACC, while nearly flat with respect to the testing data sets, is highly divergent with respect to the training data sets. The SVM model *train_50* achieves the best balanced accuracy for all nine different testing data sets. The SVM models trained on data sets *train_30* and *train_70* are worse than *train_50* by up to 8 points, which would be viewed as a significant effect in the missense mutation field, as shown in Table 1. The *train_*10 and *train_90* sets are much worse, although these are significantly more imbalanced than used in training missense mutation classifiers. In Figure 4c, the MCC of *train_50* achieves the best results for most of the testing data sets; *train_30* is just a big higher for testing at 0.2 and 0.3, and *train_70* is a bit higher at 0.9. The MCC can be as much as 10 points higher when trained and tested on balanced data than when trained on imbalanced data (*train_70*). Figure 4d shows the area under ROC cures (AUC) behaves similarly to BACC in Figure 4b. The AUC distinguishes *train_50* from *train_30* and *train_70* to only a small extent, but the difference between these curves and *train_10* and *train_90* is fairly large.

## Discussion

A common objective in bioinformatics is to provide tools that make predictions of binary classifiers for use in many areas of biology. Many techniques in machine learning have been applied to such problems. All of them depend on the choice of features of the data that must differentiate the positive and negative data points as well as on the nature of the training and testing data sets. While computer scientists have studied the nature of training and testing data, particularly on whether such data sets are balanced or imbalanced [38], the role of this aspect of the data is not necessarily well appreciated in bioinformatics.

In this article, we have examined two aspects of the binary classification problem: the source of the input data sets and whether the training and testing sets are balanced or not. On the first issue, we found that a negative data set that is more distinct from the positive data set results in higher prediction rates. This result makes sense of course, but in the context of predicting missense mutation phenotypes it is critical that the neutral data points are truly neutral. We compared the ability of primate/human sequence differences and human polymorphisms to predict disease phenotypes. The primate/human sequence differences come from a small number of animal samples and the reference human genome, which is also from a small number of donors. The majority of intraspecies differences are rare, and thus the majority of primate/human differences are likely to reflect true species differences rather than polymorphisms within each species. It seems likely that they should be mostly neutral mutations, or the result of selected adaptations of the different species.

On the other hand, the polymorphisms in the SwissVar database are differences among hundreds or thousands of human donors. Their phenotypes and prevalence in the population are unknown. It is more likely that they are recent sequence changes which may or may not have deleterious consequences and may or may not survive in the population. Some authors have tried to estimate the percentage of SNPs that are deleterious. For instance, Yue and Moult estimated by various feature sets that 33–40% of missense SNPs in dbSNP are deleterious [67]. However, the training set for their SVMs contained 38% deleterious mutations and it may be that these numbers are correlated. In our case, we predict that 40% of the SwissVar polymorphisms are deleterious, while only 20.6% of the primate mutations are predicted as deleterious. With a positive predictive value of 80.4%, then perhaps 32.4% of the SwissVar polymorphisms are deleterious.

In any case, the accuracy of missense mutation prediction that one may obtain is directly affected by the different sources of neutral data and deleterious data, separately from the choice of features used or machine learning method employed. Results from the published literature should be evaluated accordingly.

We have examined the role of balanced and imbalanced training and testing data sets in binary classifiers, using the example of missense phenotype prediction as our benchmark. We were interested in how we should train such a classifier, given that we do not know the rate of deleterious mutations in real-world data such as those being generated by high-throughput sequencing projects of human genomes. Our results indicate that regardless of the rates of positives and negatives in any future testing data set such as human genome data, support vector machines trained on *balanced* data sets rather than imbalanced data sets performed better on each of the measures of accuracy commonly used in binary classification, i.e. balanced accuracy (BACC), the Matthews correlation coefficient (MCC), and the area under ROC curves (AUC). Balanced training data sets result in high, steady values for both TPR and TNR (Figure 3a and 3b) and good tradeoffs in the values of PPV and NPV (Figure 3c and 3d).

Even at the mild levels of training imbalance shown in Table 1 (30–40% in the minority class), there would be what would be considered significant differences in balanced accuracy of about 8% and MCC of 10%. The AUC is considerably less sensitive to the imbalance in the training set from 30–70% deleterious mutation range, probably because it measures only the ordering of the predictions rather than a single cutoff to make one prediction or the other.

For the programs listed in Table 1, it is interesting to examine their efforts in considering the consequences of potential imbalance in the training data sets. The authors of both SNAP [5], [6] and MuD [7] used very nearly balanced training data sets and noted the effect of using imbalanced data sets in their papers. In MuD’s case, they eliminated one third of the deleterious mutations from their initial data set in order to balance the training data. SNSPs3D-stability [67] was derived with the program SVMLight [68]–[70], which allows for a cost model to upweight the misclassification cost of the minority class, which the authors availed themselves of. MuStab [46] also used SVMLight but the authors did not use its cost model to account for the imbalance in their training data set (31% deleterious). The program LIBSVM [71] also allows users to use a cost factor for the minority class in training. Two of the programs in Table 1, SeqProfCod [41], [45] and PHD-SNP [40] used this program, but did not use this feature to deal with imbalance in their training data sets. Finally, programs using other methods such as a Random Forest (SeqSubPred [72] and nsSNPAnalyzer [3]), a neural network (PMut [2], [43], [44]), and empirical rules (PolyPhen2 [73]) also did not address the issue of training set imbalance.

In any case, given that relatively large training and testing data sets can be obtained for the missense mutation classification problem (see Table 1), it is clear that balancing the data in the training set is the simplest way of dealing with the problem, rather than employing methods that treat the problem in other ways (oversampling the minority class, asymmetric cost functions, etc.).

In light of the analysis presented in this paper, it is useful to examine one other group of binary classifiers in bioinformatics – that of predicting disordered regions of proteins. These classifiers predict whether a residue is disordered or ordered based on features such as local amino acid composition and secondary structure prediction. However, the typical training and testing data sets come from structures in the Protein Data Bank, which typically consist of 90–95% ordered residues. Only 5–10% of residues in X-ray structures are disordered and therefore missing from the coordinates. We examined the top five predictors in the most recent CASP experiment [74] in terms of how the methods were trained and tested. These methods were Prdos2 [14], Disopred3C [75], Zhou-Spine-D [16], CBRC_Poodle [17], and Multicom-refine [76]. Some parameters of the data sets from the published papers and the prediction rates from the CASP9 results are shown in Table 5. All five methods were trained on highly imbalanced data sets, ranging from just 2.5% disordered (DisoPred3C) to 10% disordered (Zhou-Spine-D). DisoPred3C also had the lowest TPR and highest TNR of these five methods, which is consistent with the results shown in Figure 3a and 3b. It was also the only method that specifically upweighted misclassified examples of the minority class (disordered residues) during the training of a support vector machine using SVMlight, although they did not specify the actual weights used. The developers of Zhou-Spine-D used a marginally imbalanced training set to predict regions of long disorder (45% disordered), arguing that this situation is easier than predicting disorder in protein structures, where the disorder rate is about 10%. In the latter case, they use oversampling of the minority class of disordered residues in order to train a neural network. The other three methods listed in Table 5 did not use available cost models in the machine learning methods they used, including LIBSVM (CBRC-Poodle) or SVMLight (Prdos2) or any form of weighting or oversampling in a neural network (Multicom-refine). Because the percentage of disordered residues in protein structures is relatively low, it may be appropriate to apply asymmetric costs and oversampling techniques in attempting to account for the skew in training data in the disorder prediction problem, but these techniques have not been widely applied for the disorder prediction problem.

**Table 5:** Top five predictors tested by CASP9 targets (117 targets).

Predictors	Residues indata set	%Dis	TPR	TNR	BACC	MCC	AUC
PrDOS2	109921	4.8	60.7	90.1	75.4	0.418	0.855
DisoPred3C	171960	2.5	34.7	99.2	67.0	0.508	0.854
Zhou-Spine-D	933382	10.0	57.9	88.3	73.1	0.365	0.832
CBRC_Poodle	18627	6.0	44.6	94.2	69.4	0.386	0.831
Multicom-refine	201703	6.4	65.0	85.0	75.0	0.365	0.822

In summary, the problem of imbalanced training data occurs frequently in bioinformatics. Even mild levels of imbalance – at 30–40% of the data in the minority class – is sufficient to alter the values of the measures commonly used to assess performance in ways that authors of new studies would think of as notable differences. When large amounts of data in the minority class are easy to obtain, the simplest solution is to undersample the majority class and effectively balance the data sets. When these data are sparse, then bioinformatics researchers would do well to consider techniques such as oversampling and cost-sensitive learning developed in machine learning in recent years [30] [77]–[79].

## Supporting Information

Data S1 (ZIP)
